# Resection of Gliomas with and without Neuropsychological Support during Awake Craniotomy—Effects on Surgery and Clinical Outcome

**DOI:** 10.3389/fonc.2017.00176

**Published:** 2017-08-18

**Authors:** Anna Kelm, Nico Sollmann, Sebastian Ille, Bernhard Meyer, Florian Ringel, Sandro M. Krieg

**Affiliations:** ^1^Department of Neurosurgery, Klinikum rechts der Isar, Technische Universität München, Munich, Germany; ^2^TUM-Neuroimaging Center, Klinikum rechts der Isar, Technische Universität München, Munich, Germany

**Keywords:** awake surgery, brain tumor, direct electrical stimulation, interdisciplinary teamwork, intraoperative testing, neuropsychologist

## Abstract

**Background:**

During awake craniotomy for tumor resection, a neuropsychologist (NP) is regarded as a highly valuable partner for neurosurgeons. However, some centers do not routinely involve an NP, and data to support the high influence of the NP on the perioperative course of patients are mostly lacking.

**Objective:**

The aim of this study was to investigate whether there is a difference in clinical outcomes between patients who underwent awake craniotomy with and without the attendance of an NP.

**Methods:**

Our analysis included 61 patients, all operated on for resection of a presumably language-eloquent glioma during an awake procedure. Of these 61 cases, 47 surgeries were done with neuropsychological support (NP group), whereas 14 surgeries were performed without an NP (non-NP group) due to a language barrier between the NP and the patient. For these patients, neuropsychological assessment was provided by a bilingual resident.

**Results:**

Both groups were highly comparable regarding age, gender, preoperative language function, and tumor grades (glioma WHO grades 1–4). Gross total resection (GTR) was achieved more frequently in the NP group (NP vs. non-NP: 61.7 vs. 28.6%, *P* = 0.04), which also had shorter durations of surgery (NP vs. non-NP: 240.7 ± 45.7 vs. 286.6 ± 54.8 min, *P* < 0.01). Furthermore, the rate of unexpected tumor residuals (estimation of the intraoperative extent of resection vs. postoperative imaging) was lower in the NP group (NP vs. non-NP: 19.1 vs. 42.9%, *P* = 0.09), but no difference was observed in terms of permanent surgery-related language deterioration (NP vs. non-NP: 6.4 vs. 14.3%, *P* = 0.48).

**Conclusion:**

We need professional neuropsychological evaluation during awake craniotomies for removal of presumably language-eloquent gliomas. Although these procedures are routinely carried out with an NP, this is one of the first studies to provide data supporting the NP’s crucial role. Despite the small group size, our study shows statistically significant results, with higher rates of GTR and shorter durations of surgery among patients of the NP group. Moreover, our data emphasize the common problem of language barriers between the surgical and neuropsychological team and patients requiring awake tumor resection.

## Introduction

For an optimal oncological outcome after surgery, it is important to maximize the extent of resection (EOR) of low- and high-grade gliomas. Gross total resection (GTR) relates to a longer progression-free survival (1–4). It is necessary to save as much healthy brain tissue as possible to achieve this optimal oncological outcome without long-lasting deterioration of language, motor, or neurocognitive functions while preserving the greatest possible quality of life (1, 4–6). By means of preoperative imaging, brain-stimulation techniques, and functional examinations, it is feasible to reveal whether a brain lesion is located in an eloquent region, which affects the planning of the surgical procedure.

The current gold standard during resections of low-grade and high-grade gliomas that are presumably located in language-eloquent areas is awake surgery combined with intraoperative direct electrical stimulation (DES) and intraoperative neuromonitoring (IOM) (2, 3, 6–9). Intraoperative DES is used during awake craniotomy because assessing language function requires an attentive patient. For language evaluation, a neuropsychologist (NP) is commonly regarded as an essential member of an interdisciplinary surgical team. On the one hand, the NP has to support the patient during this special situation of awake surgery and has to decide whether language function is being affected by intraoperative DES or by another factor (e.g., physical or psychological stress, problems with concentration) (10, 11). Furthermore, the NP is responsible for the patient’s attention and compliance so that language function can be successfully evaluated during the awake state of surgery. On the other hand, the NP has to give prompt feedback to the neurosurgeons so that they can decide how to continue with the resection (12–14). Nevertheless, the duration of surgery should not be longer than necessary, and the neuropsychological testing procedure should give precise information about the patient’s language function (11). Yet, data regarding the actual effects of neuropsychological involvement during awake surgery are lacking.

Therefore, the aim of this study was to investigate whether there is a difference in clinical outcomes between patients who underwent awake craniotomy for glioma removal with or without the attendance of an NP due to a significant language barrier between the patient and the NP.

## Materials and Methods

### Study Design

This study analyzes a prospectively gathered, consecutive cohort on a post hoc basis. As inclusion criteria, we defined the following characteristics for our analysis:

– age above 18 years,
– written informed consent,
– glioma (WHO grades 1–4) with a left-hemispheric language-eloquent location,
– preoperative language and communication status that allowed for intraoperative language testing (according to preoperative evaluation),
– complete intraoperative language testing according to a standardized protocol (by an NP in the NP group or a bilingual resident for the non-NP group), and
– awake craniotomy for resection of the lesion at our department.

The exclusion criteria were as follows:

– preoperative aphasia not allowing for intraoperative language testing (according to preoperative evaluation),
– history of difficult intubation or chronic cough as contraindications against an awake procedure, and
– anxiety or non-compliance not allowing for awake surgery.

### Patients and Procedures

We enrolled 61 consecutive patients deemed eligible for an awake craniotomy. Fully trained and experienced neurosurgeons from our department performed all surgeries for tumor removal between 2008 and 2016. Of these 61 patient cases, 47 surgeries were done with neuropsychological support (NP group), whereas 14 surgeries were conducted without an NP (non-NP group). Regarding the patients in the non-NP group, their mother tongue and language tested during surgery was not German, thus not allowing the NP to support the surgical team during resection due to a significant language barrier. Therefore, the NP was not able to evaluate induced language impairment directly in the non-NP group. In these cases, a bilingual resident speaking the mother tongue of the respective patient carried out intraoperative language testing.

#### Preoperative Setup

Each patient underwent detailed clinical examinations within the week before surgery for tumor resection. The physical and neurological examination performed by a neurosurgical resident covered sensory function, coordination, muscle strength (according to the standardized scale of the British Medical Research Council), and cranial nerve function with respect to a standardized protocol routinely used at our department for brain tumor patients. Any deficits were recorded for later comparison to the postoperative state. We also determined the individual’s preoperative Karnofsky performance status (KPS) score. In addition, the neurosurgeon evaluated each patient’s cooperativity, power of concentration, and compliance to guarantee that the individual patient was able to undergo an awake craniotomy.

A fully trained NP tested preoperative language ability in all of the patients. For the patients in the non-NP group, a translator supported the NP during the preoperative evaluation. The NP used the Aachen Aphasia Test (AAT) to evaluate language, supplemented by further definitions of aphasia grades, which were defined as follows and applied previously by our group (15–18):

– no deficit,
– mild deficit (normal speech comprehension and/or conversational speech with slight amnesic aphasia, adequate communication ability),
– medium deficit (minor disruption of speech comprehension and/or conversational speech, adequate communication ability), and
– severe deficit (major disruption of speech comprehension and/or conversational speech, clear impairment of communication ability).

Furthermore, the NP used the following language-related tasks during the preoperative assessment (19–22):

– object naming,
– verb generation, and
– counting.

The initial magnetic resonance imaging (MRI) consisted of three-dimensional gradient echo sequences with and without the application of an intravenous contrast agent, a fluid-attenuated inversion recovery (FLAIR) sequence, and diffusion tensor imaging (DTI) sequences with respect to a standard protocol for tumor patients at our hospital. All imaging was performed on 3 T MRI scanners (Achieva 3 T, Philips Medical Systems, The Netherlands B.V., or Verio 3 T, Siemens Healthcare, Erlangen, Germany) using an 8-channel phased-array head coil. Furthermore, functional MRI sequences to map language function or positron emission tomography were added in selected cases. Gradient echo sequences were used to measure the maximum tumor diameter in all three axes. Furthermore, the tumor volume was assessed in these sequences by displaying the lesion in axial, sagittal, and coronal slices and using a smart-brush algorithm implemented in our surgical neuronavigation applications (BUZZ, Brainlab AG, Munich, Germany).

As part of the preoperative setup, the patients also underwent language mapping by navigated transcranial magnetic stimulation (nTMS) in combination with an object-naming task (eXimia, Nexstim Plc., Helsinki, Finland) (16–18, 23). All of the preoperative imaging data, including DTI-based tractography and nTMS mapping, were evaluated by an interdisciplinary tumor board and used for preoperative resection planning within a surgical neuronavigation system (Brainlab iPlan Net server, version 3.0.1, Brainlab AG, Munich, Germany) after indication for awake surgery was made and the surgery was discussed with the patient.

#### Intraoperative Setup

All of the enrolled patients underwent awake surgery for tumor removal following an asleep–awake–asleep approach (18, 24). Our protocol followed the established guidelines for awake craniotomies (20, 22, 25, 26). All anatomical and functional imaging and nTMS mapping data were available on intraoperative navigational screens (Brainlab Curve, Brainlab AG, Munich, Germany). No distinction was made between the NP group and the non-NP group regarding the surgical staff, anesthesia protocols, or surgical techniques used. The only difference between groups was that no NP was available for the patients of the non-NP group. For these patients, a resident conducted intraoperative language assessments.

In short, a combination of bupivacaine and epinephrine was used for regional and infiltration anesthesia of the galea and dura. Analgesia and sedation were achieved by continuous infusion of remifentanil and propofol. The sedation was stopped 10 min before the intraoperative language testing during the awake state. Intraoperative DES was then performed with a bipolar (cortical stimulation) or monopolar (cortical and subcortical stimulation) electrode (Inomed Medizintechnik GmbH, Emmendingen, Germany). A surface electroencephalogram was recorded to detect intraoperative seizures (27, 28).

Language mapping by intraoperative DES during the awake state of the surgery included object naming and counting, as applied during the preoperative setting (19–22). Each cortical site was stimulated in steps of 5 mm at least three times, and language-positive spots (stimulation points at which an error was elicited) were marked by a number tag on the cortex (27, 28). After the intraoperative DES was finished, resection was started under continuous monitoring of overt speech.

#### Postoperative Setup

All of the patients underwent MRI scanning on their first postoperative day to assess the EOR with the same imaging protocol as the one used preoperatively. This protocol was also repeated during routine follow-up examinations every 3–12 months, and at least two board-certified neuroradiologists evaluated the MRI scans. Unexpected residuals were present when the neurosurgeon assumed intraoperative GTR but the postoperative MRI showed clear residual tumor tissue. The volume of the residual tumor tissue (0 cm^3^ in cases where GTR was achieved) was measured and subtracted from the preoperative tumor volume to be able to provide volumetric EOR data. Furthermore, anterior–posterior (AP), lateral, and overall craniotomy sizes were measured in postoperative imaging.

The preoperative physical and neurological examinations were conducted again for each patient immediately after surgery and on a daily basis from the first postoperative day until discharge and during follow-up visits every 3–12 months, depending on the lesion’s histopathological entity. Any neurological deficits were carefully documented and compared to the preoperative state. Each patient’s KPS score was again determined on the day of discharge. Regarding language function, aphasia gradings were repeated and compared during the postoperative and follow-up examinations to determine any surgery-related deficits, as done in previous reports (17):

– surgery-related transient language impairment: any new or worse language deficits due to tumor removal that were resolved within 3 months after surgery, and
– surgery-related permanent language impairment: any new or worse language deficits due to tumor removal that did not return to the preoperative status within 3 months.

All patients of the NP and non-NP group underwent systematic neurorehabilitation with NP support starting in the hospital within the first days after surgery, and treatment was continued in certified rehabilitation facilities after discharge according to current standards (29–32).

### Statistics

Fisher’s exact tests, unpaired *t* tests, and Mann–Whitney *U* tests were performed to assess the differences between patients in the NP group and the non-NP group. First, the Shapiro–Wilk test was applied to test the normality of the data. A *P*-value <0.05 was considered statistically significant. All statistical analyses were performed using GraphPad Prism (version 7.0; GraphPad Software Inc., La Jolla, CA, USA).

## Results

### Patient Characteristics

There were no statistically significant differences between the NP group and non-NP group regarding age, gender, preoperative language deficits, tumor grade according to histopathological evaluation, or time to progression-free follow-up (Table 1). However, the maximum diameter of the lesion was larger in the non-NP group (Table 1).

**Table 1:** Characteristics of patients.

		Neuropsychologist (NP)	Non-NP	*P*-value
Number of patients		47	14	–

Age (in years, mean ± SD)		45.9 ± 14.4	40.2 ± 9.3	0.17

Gender (in %, male/female)		61.7/38.3	71.4/28.6	0.75

Preoperative language deficits (in %)	None	55.4	64.3	0.57
	Mild	19.1	28.6	
	Medium	23.4	7.1	
	Severe	2.1	0.0	

Tumor grade (in %)	I	2.1	0.0	0.59
	II	31.9	28.6	
	III	25.5	42.8	
	IV	40.5	28.6	

Maximum diameter of the lesion (in cm, mean ± SD)		3.5 ± 1.1	4.5 ± 1.7	0.02

Follow-up without progression (in months, mean ± SD)		18.1 ± 18.9	19.2 ± 18.7	0.70

*Detailed overview of the number of enrolled patients, age, gender, preoperative language deficits, tumor grade (glioma WHO grades 1–4), maximum diameter of the lesion, and follow-up examinations without progression for the NP and non-NP group. There was a statistically significant difference regarding the maximum diameter of the lesions between both groups (*P* = 0.02)*.

### Surgery-Related Characteristics

#### Craniotomy Size

The AP extent of the craniotomy was 7.3 ± 1.4 cm (range: 4.1–9.2 cm) in the group of patients with NP support and 8.3 ± 1.0 cm (range: 6.5–9.9 cm) in the non-NP group (*P* = 0.02). Furthermore, the lateral extent was 5.1 ± 2.2 cm (range: 1.8–9.1 cm) in the NP group and 4.7 ± 2.3 cm (range: 2.5–8.7 cm) in the non-NP group (*P* = 0.70). The resulting overall area of the craniotomy was 36.9 ± 17.3 cm^2^ (range: 11.7–80.1 cm^2^) in the NP group and 38.7 ± 19.2 cm^2^ (range: 18.5–75.7 cm^2^) in the non-NP group (*P* = 0.78).

#### Duration of Surgery

**Figure 1:**
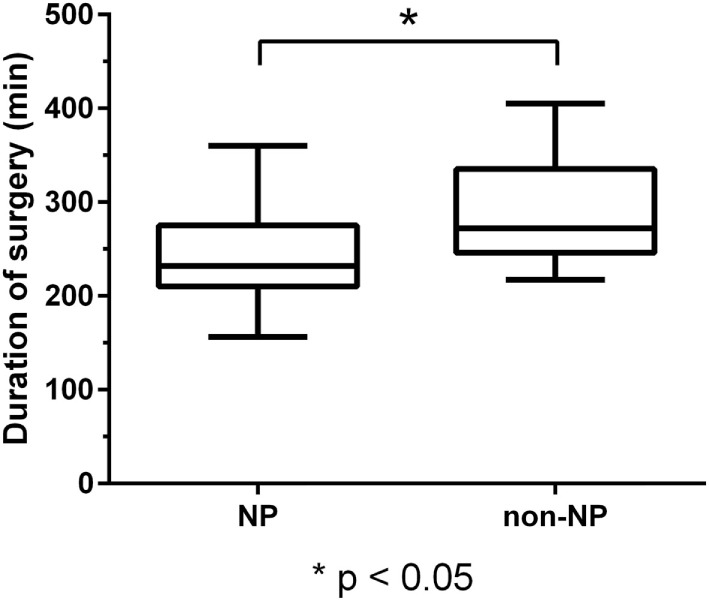
Duration of surgery. Boxplot of duration of surgery for the neuropsychologist (NP) and the non-NP group with median, minimum, and maximum whiskers and quartile-boxes. There was a statistically significant difference in the duration of surgery between both groups (*P* < 0.01).

The patient cohort with NP support during surgery showed a statistically significant shorter duration of surgery of 240.7 ± 45.7 min (range: 156.0–360.0 min), compared to 286.6 ± 54.8 min (range: 217.0–405.0 min) in the non-NP group (*P* < 0.01; Figure 1).

#### Residual Tumor

**Figure 2:**
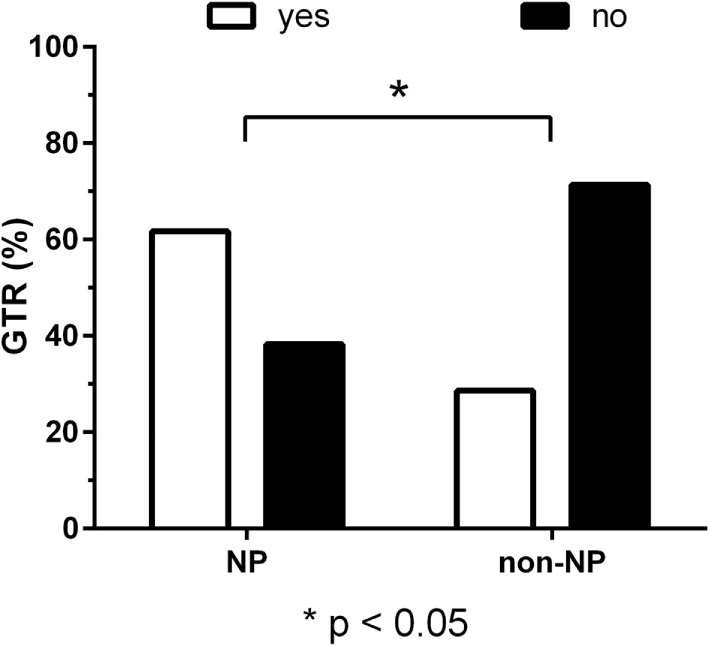
Residual tumor. Bar chart of gross total resection (GTR, in %) for the neuropsychologist (NP) and non-NP group. GTR was achieved in 61.7% of patients in the NP group and 28.6% of patients in the non-NP group according to magnetic resonance imaging (MRI) performed after surgery (*P* = 0.04).

According to the neurosurgeon’s intraoperative estimation, GTR was achieved in 38 patients (80.9%) of the NP group and in 10 patients (71.4%) of the non-NP group (*P* = 0.47). Furthermore, unexpected residuals (estimation of the intraoperative EOR vs. postoperative imaging) were found in nine patients (19.1%) of the NP group and in six patients (42.9%) of the non-NP group. However, this difference between groups was not statistically significant (*P* = 0.09). Accordingly, residual tumor tissue was found in the postoperative MRI of 18 patients (38.3%) of the NP group and in 10 patients (71.4%) of the non-NP group (*P* = 0.04; Figure 2).

Among patients of the NP group, the initial brain lesion showed a mean volume of 34.8 ± 21.0 cm^3^. The volume of the tumor residual accounted for 1.5 ± 1.8 cm^3^, which implies that 95.7% of the initial volume were resected. Concerning patients of the non-NP group, the preoperative tumor volume was 43.8 ± 40.9 cm^3^, whereas the postoperative residual volume was 1.4 ± 1.2 cm^3^. Accordingly, 96.8% of the preoperative tumor volume were surgically removed. Regarding the difference in residual tumor volumes between the NP and non-NP group, no statistically significant difference was revealed (*P* = 0.97).

### Clinical Course and Functional Outcome

#### Karnofsky Performance Status

There were no statistically significant differences between both groups in terms of their preoperative or postoperative KPS scores. The median preoperative KPS score was 90 (range: 60–100) in the NP group and 100 (range: 80–100) in the non-NP group (*P* = 0.05). The median postoperative KPS score was 90 (range: 10–100) for the NP group and 90 (range: 60–100) for the non-NP group (*P* = 0.41).

Furthermore, the median KPS score during the follow-up examinations was 90 (range: 0–100) in the NP group and 90 (range: 60–100) in the non-NP group. This difference in follow-up KPS scores was not statistically significant (*P* = 0.85). When comparing the difference between preoperative and follow-up KPS scores, there was again no statistically significant difference between patients of the NP and non-NP group (*P* = 0.05). Accordingly, patients of the NP group showed a median in change of 0 (range: −90 to 10) between preoperative and follow-up examinations, whereas patients of the non-NP group decreased by a median of 5 (range: −30 to 0).

#### Language and Other Neurological Function

**Figure 3:**
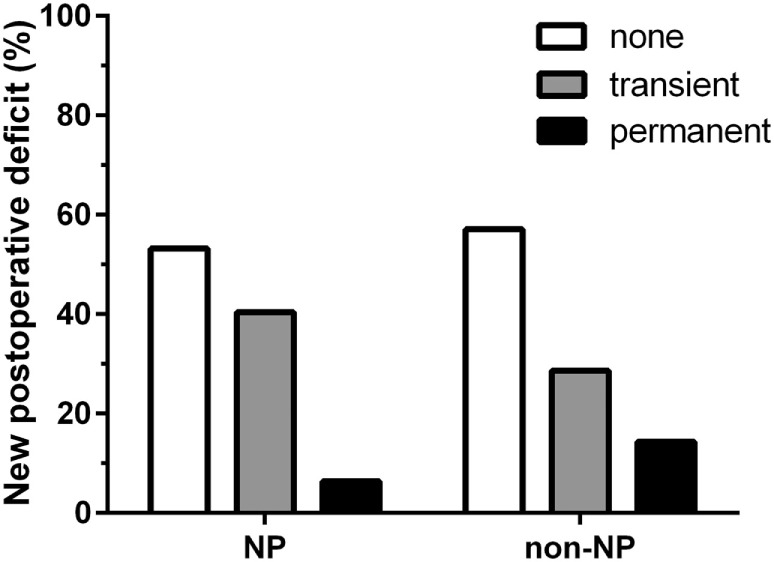
Surgery-related language deterioration. Bar chart comparing surgery-related language worsening between the neuropsychologist (NP) and non-NP group. In the NP group, 53.2% of patients showed no new surgery-related deficits, whereas transient deficits occurred in 40.4% of the patients and permanent deficits occurred in 6.4% of the patients. In the non-NP group, no new surgery-related deficits were documented for 57.1% of patients, whereas 28.6% of the patients were diagnosed with transient deficits and the remaining 14.3% of the patients suffered from permanent deficits. Regarding surgery-related permanent deficits, there was no statistically significant difference between groups (*P* = 0.48).

Regarding the status of language function, there were no statistically significant differences between groups regarding their preoperative (*P* = 0.57), postoperative (*P* = 0.22), or follow-up examinations (*P* = 0.85). Furthermore, new surgery-related permanent deficits occurred in three cases (6.4%) of the NP group and in two cases (14.3%) of the non-NP group (*P* = 0.48; Figure 3). Results regarding further neurological outcome can be found in Table 2.

**Table 2:** Neurological function.

		Neuropsychologist (NP)	Non-NP	*P*-value
Epileptic seizures (in %)	Preoperative	70.2	78.6	0.74
	Postoperative	76.6	78.6	1.00
	Follow-up	14.9	0.0	0.19

Motor deficits (in %)	Preoperative	10.6	7.1	1.00
	Postoperative	19.1	21.4	1.00
	Follow-up	17.0	14.3	1.00

Hypesthesia (in %)	Preoperative	6.4	14.3	0.32
	Postoperative	12.8	14.3	1.00
	Follow-up	10.6	0.0	0.58

*Detailed overview of the percentage of patients who showed epileptic seizures, motor deficits (according to the scale of the British Medical Research Council; a score of <5/5 in any of the muscles of the upper or lower limb was defined as a motor deficit), and hypesthesia during preoperative, postoperative, and follow-up examinations. There were no statistically significant results observed between the NP and non-NP group*.

## Discussion

The current gold standard for resection of gliomas located in language-eloquent brain regions is awake surgery combined with intraoperative DES and IOM (2, 3, 6–9). Previous studies have shown that awake craniotomy is a safe procedure for resection of lesions that are located in or close to regions of highly eloquent brain functions (1, 33, 34). Moreover, it has been demonstrated that intraoperative DES can maximize the EOR and, at the same time, contributes to the preservation of function (1, 4–6). Thus, the rationale for performing awake procedures is to achieve optimal oncological outcomes that are related to longer overall survival, longer progression-free survival, and reduced malignant transformation of low-grade gliomas (1, 3, 4).

In the last decades, the awake craniotomy procedure—which includes the surgical technique itself, the intraoperative process of mapping and language assessment, and the anesthesia protocol—was further improved (35). To minimize the incidence of failures during awake craniotomy, a professional preoperative evaluation by a multidisciplinary team that includes careful selection of patients should be considered standard (2, 10, 36, 37). This multidisciplinary team should not only include medical doctors; instead, an NP is considered a highly valuable member who is at least partially responsible for neuropsychological and neurocognitive patient assessment and selection of patients suitable for awake surgery. In this context, the importance of the preoperative selection process for patients who might be suitable for an awake procedure and the detailed information given to these patients about the upcoming surgery have already been described (11, 38). Overall, a thorough preoperative assessment can increase the patients’ compliance and reduce fear before and during surgery (11, 38).

The aim of our study was to demonstrate the impact of a professional evaluation of language function by an NP during intraoperative DES on surgical and functional outcomes. Most large and specialized departments perform awake surgeries for tumor resection in close collaboration with an NP or speech therapist for pre- and perioperative language evaluation (2, 6, 9). Yet, the necessity of having an NP present during awake surgery has not yet been shown scientifically, and financing this additional staff can be difficult in times of increased health care costs and case-based reimbursements. To the best of our knowledge, only one other published study has described a better functional outcome when a specialized therapist monitored neurological functions during intraoperative mapping (39). However, a group-comparison analysis that provides data showing the necessity of NP support has been missing. Likewise, no data were available investigating the impact of language barriers in awake surgery although many of us face this quest quite regularly.

Patients of the NP and non-NP group were highly comparable except for tumor size based on the maximum diameter (larger in the non-NP group). The surgical technique and team were identical in both groups. We found that the duration of surgery in the NP group was significantly shorter when compared to the non-NP group (Figure 1). Possible causes for the longer durations in the non-NP group might include the need for translation, insufficient routines when replacing the NP, insecurity concerning induced language impairments, and generally less confidence in the mapped functional anatomy by the surgical team, thus requiring extended testing during a prolonged awake phase. Furthermore, GTR was more frequently found in the NP group (Figure 2). If we assume that the evaluation of language function during intraoperative DES is more reliable and straightforward when performed by an NP, we can expect that the reactions of and the confidence in the following intraoperative decisions made by the surgeon had a direct influence on the EOR. Thus, the surgeon feels more confident and is able to perform a more aggressive resection. In this context, it is well known that valid evaluations of language and, thus, of optimally performed intraoperative DES increase the opportunity for a more aggressive resection of the tumor without loss of language function, even if the lesion is considered highly eloquent (13, 14, 37). However, although we found differences in the frequency of GTR, we did not observe a difference in new surgery-related permanent language deficits between the groups (Figure 3).

Although this work focuses on the impact of NP support on parameters related to the surgical procedure, we have to keep in mind that the role of the NP is clearly not limited to intraoperative support. Instead, the NP also contributes to the pre- and postoperative setting, which even goes beyond the time of inpatient care. Regarding the preoperative phase, the NP is strongly involved in patient selection and preparation prior to awake surgery as aforementioned. Such preoperative NP support can increase the overall success of surgery by increasing compliance and reducing fear (11, 38). Concerning the postoperative phase, the NP can overtake or guide important aspects of neurorehabilitation, which have to be considered important parts of modern neuro-oncological treatment concepts (29, 30). In this context, conventional rehabilitation therapy and special neuropsychological or cognitive training have repeatedly shown to result in better outcome and enhanced cognitive performances in patients suffering from brain tumors (40–42). Since all patients enrolled in this study underwent neurorehabilitation, the comparatively low permanent deficit rates determined during follow-up examinations may at least partially be due to effective rehabilitation treatment by NPs and other caregivers and are not only the result of careful surgical resection applying intraoperative DES and IOM. Thus, future research should further investigate the important role NP support plays in terms of both pre- and postoperative aspects among neurosurgical patients.

The difference in size of the two cohorts that were compared (NP vs. non-NP) reflects the main limitation of this study. Because NP support is regarded as the clinical standard in most specialized neuro-oncological centers worldwide, it is difficult to enroll high numbers of patient cases that were operated on without such support. Hence, multi-center studies are needed to form larger cohorts based on the results shown in this work.

In summary, we were able to show statistically significant differences between both groups even though the non-NP group was comparatively small. This proves the strong effects of professional NP support on surgery and treatment. Moreover, it also shows that we need to find solutions to improve the care of patients having a significant language barrier in relation to the neuro-oncological team. While the results of this study are in accordance with the experiences of many neurosurgeons performing awake craniotomies, the data might help to emphasize the need for NP support. Our study should also strengthen the position of neuro-oncological centers toward health care providers and hospital administrators by providing further evidence to include NPs as members of the intraoperative team.

## Conclusion

Our data emphasize not only the need for professional NP attendance during the resection of presumably language-eloquent tumors in the context of awake craniotomies but also the problems we face when dealing with patients of different languages. We have shown that reliable intraoperative evaluation of language performance by an NP proficient in the mother tongue of the patient has a positive influence on the rate of GTR and can significantly reduce the overall duration of surgery. Thus, our data will further justify professional and experienced intraoperative language evaluation by an NP and also nurture the discussion on how to deal with patients having a language barrier to the surgical and neuropsychological team since this is a problem many of us face regularly.

## Availability of Data and Material

All data used for analysis are presented in the manuscript. The discussion and conclusions only rely on the data presented. Raw data can be provided upon request.

## Ethics Approval and Consent to Participate

This study was approved by the local ethics committee (registration number: 2793/10) according to ethical standards of the Declaration of Helsinki. All of the patients were informed in detail about the awake surgery and necessary examinations. Written informed consent was given prior to the surgery.

## Author Notes

All authors are strongly involved in the treatment of brain tumors, including awake surgery, preoperative mapping, and IOM in a specialized neuro-oncological center. SK is an assistant professor and attending neurosurgeon. BM is chairman and FR is vice chairman of the department.

## Author Contributions

AK, NS, SI, BM, FR, and SK were responsible for data acquisition, handled the acquired data, conducted statistical analysis, and performed literature research. AK, NS, and SK drafted the manuscript and its final revision. NS, FR, and SK are responsible for concept and design. All authors approved and corrected the final version of the manuscript.

## Conflict of Interest Statement

SK and FR are consultants for Brainlab AG (Munich, Germany). SK is a consultant for Nexstim Plc. (Helsinki, Finland). The authors do not report any conflict of interest concerning the materials or methods used in this study or the findings specified in this manuscript.

## Abbreviations

AAT, Aachen Aphasia Test; AP, anterior–posterior; DES, direct electrical stimulation; DTI, diffusion tensor imaging; EOR, extent of resection; FLAIR, fluid-attenuated inversion recovery; GTR, gross total resection; IOM, intraoperative neuromonitoring; KPS, Karnofsky performance status; MRI, magnetic resonance imaging; NP, neuropsychologist; nTMS, navigated transcranial magnetic stimulation.
